# The relationship and mechanism of screen time and academic performance among adolescents: an empirical study based on CEPS

**DOI:** 10.3389/fpubh.2025.1533327

**Published:** 2025-07-02

**Authors:** Xiaosu Feng, Shuanquan Ren, Peng Shi

**Affiliations:** ^1^School of Physical Education, Liaoning Normal University, Dalian, China; ^2^Northeast Petroleum University, Daqing, China; ^3^School of Physical Education, Shandong University of Technology, Zibo, China

**Keywords:** screen time, academic performance, screen exposure, cognition, teenagers

## Abstract

**Objective:**

There is an association between adolescents’ screen time and academic performance, but the conclusions remain controversial. In particular, there is a lack of research on Chinese students who face higher academic pressure. Based on this, we aim to explore the relationship and mechanisms between screen time and academic performance among Chinese adolescents.

**Methods:**

17,150 junior middle school students from China Education Panel Survey (CEPS) from 2013 to 2014 were selected as the participants of the survey. Multiple linear regression analysis was used to explore the relationship between screen time and adolescents’ academic performance. On this basis, the *Z*-test of regression coefficient is used to further investigate whether there is heterogeneity in the type of screen exposure in this relationship. The possible mediating mechanism of the relationship between screen time and adolescents’ academic performance was investigated through path analysis.

**Results:**

Screen time and Chinese (β = −0.022), mathematics (β = −0.048), English (β = −0.043) and 3 total scores (β = −0.113), there was a significant negative correlation (*p* < 0.01). The time spent watching TV and the time spent on the Internet and playing games were significantly negatively correlated with the academic performance of adolescents (*p* < 0.01), but there was heterogeneity in the type of screen exposure. The time spent on the Internet and playing games had a significant impact on adolescent mathematics (β = −0.063), English (β = −0.047) and total scores (β = −0.148) produced more negative effects. Path analysis found that cognitive performance was the main path of the relationship between the two, sleep time, mental health, classmate relationship and parent–child relationship were important distal paths, and social relationship construction played a stronger intermediary role in the relationship between screen time and adolescent academic performance.

**Conclusion:**

The results support the “negative impact” hypothesis, and the negative effect of surfing the Internet and playing games on adolescents’ academic performance is greater. Screen time directly or indirectly affects academic performance by increasing sleep time, mental health, classmate relationship and parent–child relationship, but the mediating effect of BMI and subjective health perception has not been verified.

## 1 Introduction

The education and health of teenagers is related to the quality of the future human resource pool of the country. The adolescent stage is a critical period for the improvement of cognitive function and the development of healthy behaviors, which will be related to their future academic achievement and physical health (1). However, with the new rapid development of screen media technology, the time of adolescent screen exposure has gradually increased, gradually causing social concern. From 2021 to 2024, the average screen time of children and adolescents in the United States increased by 49 min (2). In addition, the average daily screen time for primary, middle and high school students in China is 87 min, 115 min, and 142 min, respectively, with an increasing trend of Internet access, video games and computer use (3, 4). In particular, the COVID-19 epidemic in recent years has forced education to move from offline to online, accelerating the popularity of screen media and increasing the amount of sedentary screen time for adolescents (5). Excessive screen exposure will deprive adolescents of opportunities to engage in physical activity, increase their likelihood of myopia or astigmatism, lead to lower sleep quality, increase the risk of diseases such as obesity and hypertension, and even develop mental health problems such as anxiety and depression (6–10). Based on this, physical activity guidelines for children and adolescents in some countries have also proposed a limit of “<2-h of leisure screen time” (11, 12) and China has also proposed an initiative of “no more than 1-h cumulatively per day” (13).

A growing body of evidence (14, 15) suggests that screen exposure plays a key role in adolescent cognitive and academic performance. However, the results of related studies are not consistent, and there are mainly “positive impact” (16, 17), “negative impact” (18–21), “irrelevant” (22, 23) and “inverted U-shaped relationship” (24–26). The reasons for this are fourfold: Firstly, the content and task of screen exposure may play a moderating role in the relationship between the two. Passive television viewing processes do not require information receiver interaction; whereas Internet surfing and game playing require receiver control of the information and responding to it (27), which may have inconsistent effects on cognition. In addition, moderate intensity screen media tasks help promote cognitive development compared to high and low intensity tasks (24). Secondly, in terms of measurement instruments, researchers (28–30) often use standardized academic measures, norm-referenced tests, and logical reasoning tests to assess adolescents’ academic performance, and there may be differences in the demands of these instruments on brain cognition. Thirdly, in the data analysis, the researchers ignored the confounding effects of more confounding factors, such as socioeconomic status, homework load, birth weight, and sleep, when exploring the relationship between screen time and academic performance. Related studies (31–34) have confirmed that socioeconomic status, homework burden, birth weight, and sleep are closely related to adolescents’ academic performance. If these confounding factors are not controlled, the research results may be inaccurate. Finally, in terms of social context, there are inconsistencies in the socio-cultural environment of domestic and international studies, and differences in educational concepts and investment in education (35). Even in China, disparities in the degree of socioeconomic development and urbanization may lead to inconsistencies in the development of education in the eastern, middle and western regions of the country (36).

Compared with Western adolescents, Chinese adolescents face a larger academic burden, and many parents enhance academic performance by hiring tutors or requiring their children to attend tutoring classes (37), but the relationship between screen time and adolescent academic performance in this context is still lacking. Improvements in adolescent academic performance are associated with improvements in cognitive functions such as attention, memory, thinking, and executive functions, and executive functions in particular are considered by researchers to be a predictor of academic performance as an alternative to IQ (38). The complexity of cognitive processes dictates that adolescent academic performance promotion is a systematic project, but in terms of exploring the mechanisms of the relationship between the two, most studies (39–41) have tested the pathway hypothesis based on cognitive performance, and few studies have taken an integrative perspective, examining the role of cognitive, health, and social relationship factors more broadly.

Based on this, the study used data from the China Education Panel Survey (CEPS) to explore the relationship and possible mechanisms between screen time and academic performance of Chinese adolescents, and to analyze the relationship between different types of screen time and adolescents’ academic performance. On the one hand, this helps to enrich the research on screen time and academic performance, resolving potential discrepancies in previous studies. On the other hand, it provides evidence-based support for practical interventions aimed at promoting the healthy physical and mental development of adolescents.

## 2 Literature review and research hypothesis

### 2.1 The relationship between screen time and academic performance

Academic performance is the main indicator of the extent to which adolescents receive schooling, and it is not only the main goal of student development, but also the goal pursued by teachers, parents, and other communities of interest. The relationship between screen time and academic performance is one of the key topics of research in international education and public health.

In the era of digital media, adolescents are spending more and more time on screens and screen exposure is becoming a global concern. Does screen time play a beneficial or detrimental role in the academic performance of adolescents? This issue has been of great interest to researchers, but has not received consistent conclusions. Most studies (18–21) support the idea of “negative impact” and suggest that screen time is negatively associated with cognitive and academic performance in adolescents. For example, Yan et al. (18) showed that time spent watching television and using social networking sites during school was negatively associated with adolescents’ academic performance. Poitras et al. (20) also showed a significant adverse association between screen time and cognitive and academic performance of preschool children. Substitution theory (42) suggests that too much screen time crowds out active social and educational activities for adolescents and also leads to fewer opportunities to engage in physical activity. However, some studies (43, 44) have shown a positive association between physical activity and academic performance of adolescents. Some researchers (22, 23) also support the “non-association” perspective. Morita et al. (22) found that excessive screen time is associated with lower academic performance in males, but this association is not significant among female adolescents. Adelantado-Renau et al. (23) also demonstrated in their systematic review that there is no significant association between screen time and academic performance in children and adolescents.

However, several studies have reported positive associations between screen time and cognitive and academic performance in adolescents. For example, some studies (16, 17) suggest that active screen time such as online educational learning and puzzle games have potential benefits for cognitive and academic performance, but passive television viewing may be detrimental to cognitive functioning. The reason for the inconsistent findings may be related to the content and tasks of screen exposure, which require individuals to receive and control information for interaction, are more likely to promote cognitive development (27). It has also been suggested (24, 25) that screen exposure does not uniformly impair or promote cognitive development, but rather that there is a tipping point between the two. Moderate intensity media multitasking is associated with optimal levels of cognitive control such as filtering distracting stimuli and refreshing working memory information (26). However, this “inverted U-shaped relationship” is derived from a small sample of computer program tests and there is insufficient evidence of a non-linear relationship with academic performance of adolescents. In addition, Vohr et al. (41) have highlighted the heterogeneity of the association between the type of screen exposure and academic performance. Adelantado-Renau et al. (23) showed that television viewing was negatively associated with language, mathematics, and overall academic achievement among children and adolescents, while playing video games was only negatively associated with overall academic achievement, and the magnitude of this relationship was slightly lower than that of television viewing.

Based on this, this study proposes hypothesis H1: Screen time is negatively associated with academic performance of adolescents, and there is heterogeneity in the type of screen exposure in this relationship.

### 2.2 Mechanisms of the relationship between screen time and academic performance

**Figure 1:**
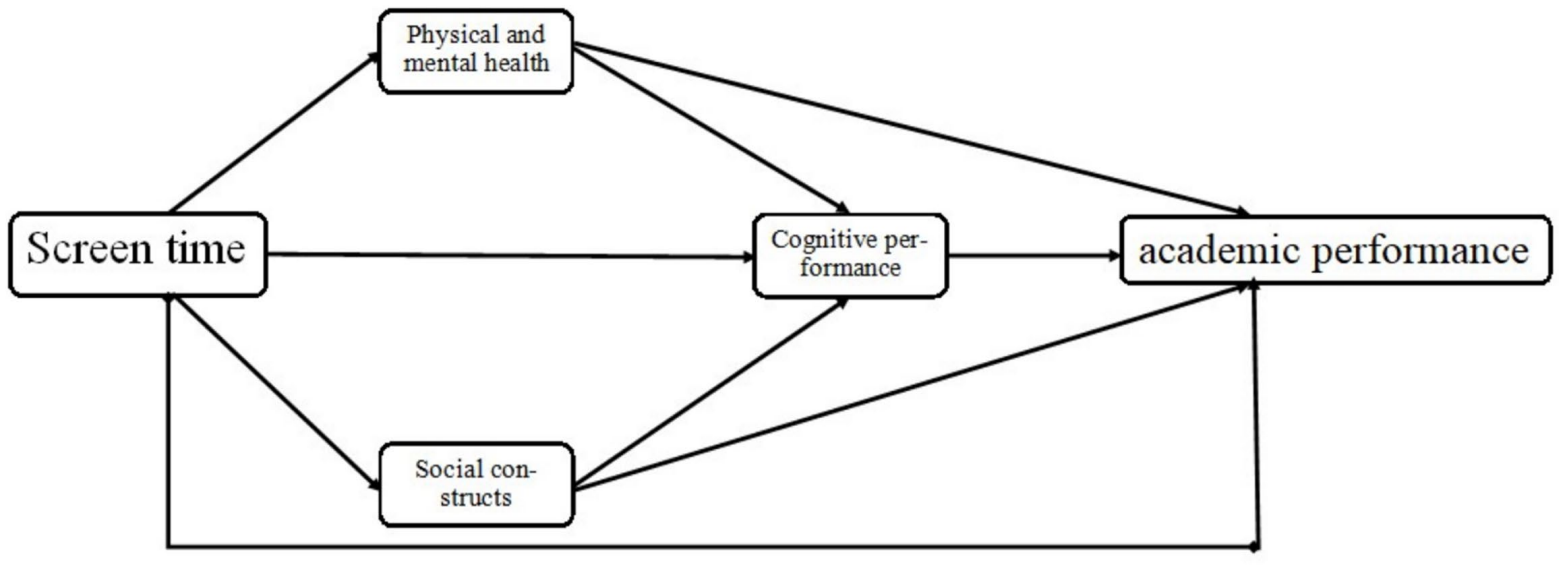
A hypothetical mechanistic model of the relationship between screen time and academic performance of adolescents.

What exactly are the factors that mediate the relationship between screen time and academic performance? No studies have been conducted to answer systematically. Wang (45) showed that screen time is the opposite of physical activity in terms of health benefits for adolescents. Although this study has not retrieved a mechanistic model of the relationship between screen time and academic performance, hypotheses can be formulated from a pathway model of the relationship between physical activity and academic performance. Tomporowski et al. (46) proposed a multi-pathway model of physical activity and cognitive and academic performance in children and adolescents, including health, physical fitness, and psychosocial factors. Based on this, Chen et al. (47) proposed a chained multi-path model of physical activity influencing academic performance in children and adolescents and emphasized cognition as a proximal predictor variable of academic performance. Therefore, based on the hypothesis that cognition is a proximal predictor variable of adolescents’ academic performance, this study followed the analytical model of sports science that explores the relationship between things mainly from the perspective of physical and mental health and social constructs, and constructed a hypothetical model of the mediating role of physical and mental health, social constructs, and cognitive performance (H2; Figure 1) to examine the mechanisms of the relationship between screen time and adolescents’ academic performance using an integrative perspective.

At the level of cognitive performance, excessive screen exposure causes a decrease in the concentration of brain-derived neurotrophic factors (48), leading to gray and white matter atrophy in children (49), reducing the functional connectivity of brain areas such as computing and reading and other areas of the brain in children (15, 50) which in turn impairs the cognitive performance of individuals (51). Johnson et al. (52) showed an association between frequent television viewing in adolescents and their future inattention and learning difficulties. Zimmerman et al. (53) showed that an average of 1 h of screen time per day for preschoolers would have negative effects on digit span memory and reading comprehension. McHarg et al. (54) and Parry and le Roux (17) showed that excessive screen media use can lead to a decrease in the level of executive function. Based on this, this study proposes research hypothesis H2a: Screen time acts on adolescents’ academic performance through cognitive performance.

At the level of physical and mental health, early and excessive exposure to electronic screens will not be conducive to the healthy physical and mental development of adolescents. Physiologically, frequent use of electronic devices not only increases the likelihood of myopia in adolescents, but also increases the risk of overweight/obesity due to sedentary behavior (55, 56). However, overweight/obesity promotes the secretion of multiple inflammatory factors in the body, regulates inflammatory signaling pathways, activates nerve cells, triggers neuro-inflammation, and subsequently affects brain function, leading to cognitive impairment (57). Related studies (58, 59) have also shown an association between overweight/obesity and lower executive function. In addition, van der Schuur et al. (60) and Ye et al. (61) showed that screen time was positively associated with sleep disturbance in adolescents. Whereas sleep is critical in maintaining normal development, neuroplasticity, and language development, adolescents with sleep disorders are more behind in cognitive performance such as memory, thinking, and executive function (62). Psychologically, studies (63–65) have shown that excessive use of digital media increases the chances of developing depression and anxiety disorders and leads to a decrease in self-esteem and subjective well-being. In particular, adolescents with depressive disorders are associated with cognitive symptoms such as inattention, memory loss, and impaired executive function (66, 67). Based on this, this study proposes research hypothesis H2b: Screen time acts on adolescents’ cognitive and academic performance through physical and mental health.

At the level of social construction, when adolescents participate in physical activities, especially in group-based programs or sports games with specific activity rules, individuals assume certain role responsibilities in the team and need to interact and cooperate with team members in order to accomplish their goals, which helps to improve self-efficacy, increase social interactions and team identity, which in turn leads to the derivation of this positive psychosocial experience into classroom learning behaviors and thus improves academic performance (68, 69). However, excessive screen exposure loses opportunities for physical activity and socialization, may cause social difficulties and social anxiety for adolescents (70), is prone to loneliness (71), and negatively affects adolescents’ social competence and behavioral problems (72), which is detrimental to the construction of social relationships. In addition, a good parent–child relationship has a positive effect on preventing undesirable behaviors and improving academic performance (73, 74), but too much screen time may lead to a weak emotional connection with each other (75). Based on this, this study proposes research hypothesis H2c: Screen time acts on adolescents’ cognitive and academic performance through social constructs.

## 3 Methods

### 3.1 Data

Participants for this study were obtained from CEPS data designed and implemented by the China Survey and Data Center at Renmin University of China. Ethical approval for the CEPS was given by the Institutional Review Board at the Renmin University of China and informed consent from research participants was obtained before the survey. The survey uses a hierarchical, multi-stage probability proportional to size (PPS) sampling method based on the order of county, school, and classroom, and aims to provide nationally representative multi-level basic data support for relevant academic research and policy formulation. The data that has been released so far includes data from the 2013 ~ 2014 academic year and the 2014 ~ 2015 academic year. Participants for the 2013 ~ 2014 academic year were sourced from 28 county-level units, 112 schools, 438 classes, and approximately 20,000 middle school students, including 10,279 7th graders and 9,207 9th graders, sampled nationwide. Participants for the 2014 ~ 2015 academic year were tracked for the first round of the survey, and a total of 9,447 students were successfully tracked and 471 new students were enrolled. This study used survey data from 2013 ~ 2014 for the follow-up empirical study because it included a sample of 7th and 9th grade students, which is a large and more representative data set. In addition, CEPS mainly focuses on the key transition stages of junior school for its investigation, and students in Grade 7 and Grade 9 are exactly at the crucial stages of transition. Among them, Grade 7 students have just stepped into junior school from primary school and are faced with challenges such as environmental adaptation, academic pressure, and the reconstruction of peer relationships. Grade 9 students, on the other hand, are at the critical point of the senior school entrance examination-based streamlining, and their academic performance directly affects their senior high school entrance pathways. During the data collection process, CEPS first obtained informed consent from the schools, and then obtained informed consent from the participants and their guardians on this basis.

**Figure 2:**
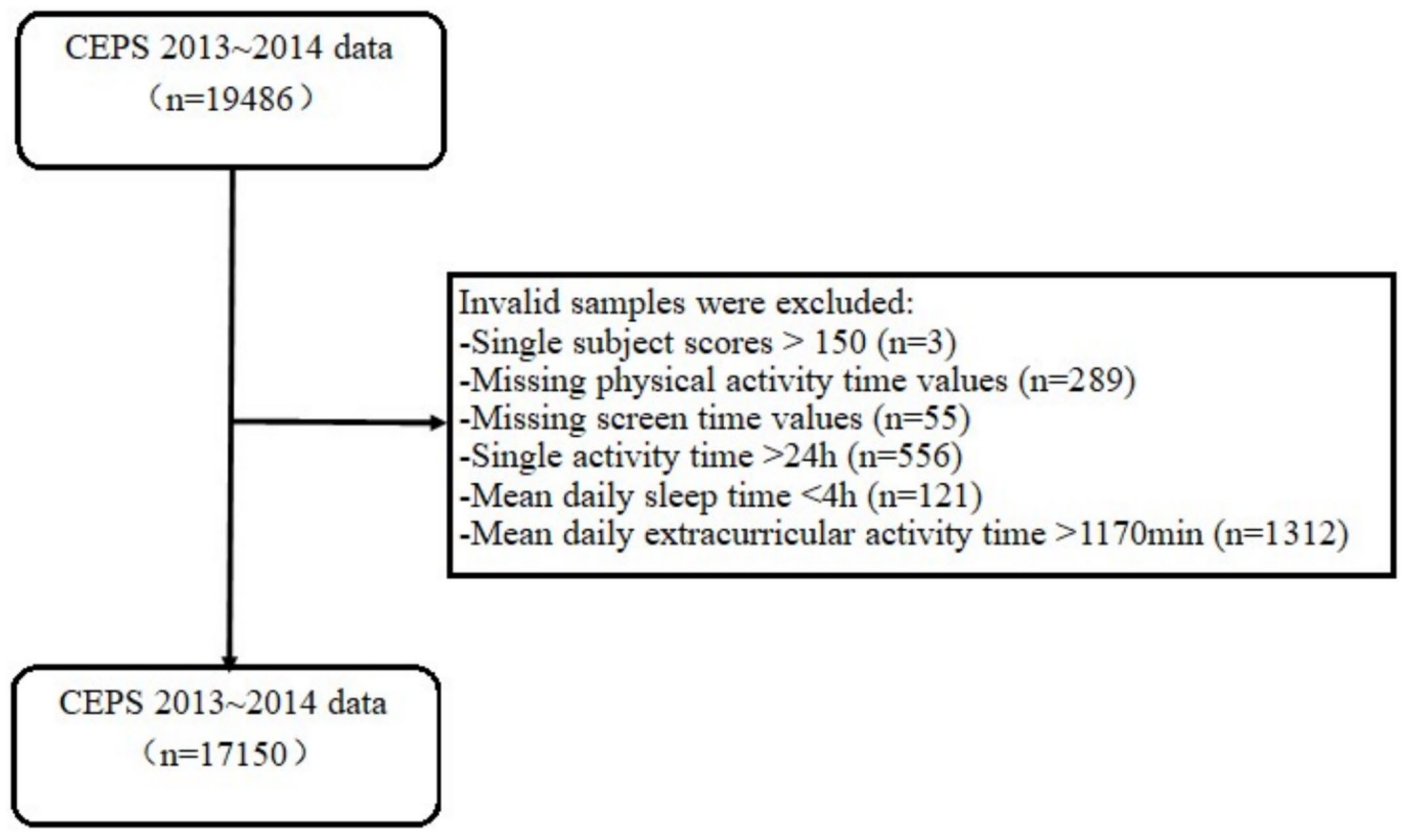
Sample screening flow chart.

To ensure the accuracy of the study results, participants were screened in this study according to the following process. Firstly, the maximum score for each subject of Chinese, Mathematics, and English is 150 points. Therefore, it is necessary to exclude participants whose single-subject scores exceed 150 points, which may be due to data entry errors. Secondly, physical activity is closely related to academic performance (19, 29, 43), which may interfere with the relationship between screen time and academic performance. Therefore, it is necessary to control for this in the analysis. Based on this, participants with missing data on physical activity time on school days and rest days were excluded. Thirdly, participants with missing values for time spent watching television, surfing the Internet and playing games on school days and rest days were excluded. Fourthly, participants with an average daily single activity time greater than 24 h were excluded. A single activity time exceeding 24 h per day suggests the possibility that participants may have been answering the questionnaire indiscriminately. Fifthly, generally speaking, the sleep duration for junior high school students should be maintained at 7 ~ 8 h. However, according to the data from CEPS, it was found that some participants had sleep durations below 7 h. Therefore, combining expert consultation and analysis of outliers, participants with sleep durations below 4 h were identified as having filled in the information indiscriminately and were excluded. Sixthly, the average middle school student has 6 classes per day, 45 min per class, so the average daily class time is 270 min. If physical exercise, screen exposure, sleep, tutorial classes, and homework time exceed 1,170 min, they will be excluded. Ultimately, this study obtained a valid sample of 17,150 cases (Figure 2). After sample screening, this study used linear interpolation to fill in the missing values.

### 3.2 Variables and instruments

#### 3.2.1 Dependent variables

The dependent variable of this study was the adolescents’ academic performance. CEPS obtained the participants’ Chinese, Mathematics, and English scores from the midterm exams in the fall semester of the 2013 ~ 2014 academic year, with a full score of 150 points for each subject. In this study, the academic performance of adolescents was reflected by middle school students’ performance in Chinese, Mathematics and English as well as their overall performance in the three subjects.

#### 3.2.2 Independent variables

The independent variable in this study was adolescents’ average daily screen time. The CEPS surveyed students’ screen time during school days by asking, “From Monday to Friday last week, how many hours and minutes on average did you spend watching TV each day?” and “From Monday to Friday last week, how many hours and minutes on average did you spend on the internet or playing video games each day?” It also surveyed students’ screen time on weekends by asking, “On the weekend last week, how many hours and minutes on average did you spend watching TV each day?” and “On the weekend last week, how many hours and minutes on average did you spend on the internet or playing video games each day?” Participants were required to report the average number of hours and minutes they spent watching TV, using the internet, and playing video games each day during school days and on weekends based on their actual experiences. This study aggregated these into total weekly screen exposure in minutes and then divided the total by 7 to obtain the average daily screen time. In addition, the relationship between non-interactive television watching and interactive Internet surfing and game playing with adolescents’ academic performance was also explored separately. The subjective measurement items based on questionnaires in CEPS mainly originate from the Chinese version of the Children’s Leisure Activities Study Survey (CLASS-C) (76). CLASS-C primarily investigates the types and duration of screen time among Chinese children and adolescents and has been proven to have high reliability and validity (76).

#### 3.2.3 Mediating variables

This study focuses on mediating variables at the cognitive performance, physical and mental health, and social construct levels.

Firstly, the cognitive test of CEPS is an international standardized cognitive assessment test, which mainly examines adolescents’ logical thinking and problem solving ability, including 3 dimensions and 11 constructs of language, graphics, computation and logic, and the scores take the value of 0 to 22 (77). The test does not examine specific literacy knowledge taught in the school curriculum and is internationally comparable and nationally standardized (78).

Secondly, the variables of adolescents’ physical and mental health include body mass index (BMI), subjective health perceptions, sleep duration, and mental health. BMI was obtained by calculating weight (kg)/height (m)^2^. Subjective health perceptions were assigned using a 5-point scale, with 1 to 5 indicating very unhealthy to very healthy. This study obtained the average minutes of sleep per day by calculating the average hours and minutes of sleep per day. This study used exploratory factor analysis to extract one composite indicator from the five indicators reflecting adolescents’ mental health (frustration, depression, sadness, unhappiness, and life is not fun), with higher values indicating lower mental health.

Finally, the variables of adolescents’ social constructs include peer relationships and parent–child relationships. The CEPS reflects peer relations with the statement “Most of my classmates are friendly to me” and is assigned on a 4-point scale, with 1 to 4 indicating complete disagreement to complete agreement. The CEPS used the questions “How is your relationship with your father?” and “How is your relationship with your mother?” to reflect father-child and mother–child relationships. The above two items were designed with a 3-point self-rating scale, requiring participants to choose the appropriate option based on their actual situation. This study employed exploratory factor analysis to extract a composite indicator from the above two questions to reflect parent–child relationships, with higher values indicating closer relationships.

#### 3.2.4 Control variables

This study referred to similar studies (28, 75, 79), and a total of 18 control variables were selected at 3 levels: individual, family, and school, based on the levels of investigation of the CEPS.

The individual level includes six variables: grade, gender, ethnicity, household registration, birth weight, and myopia. Ethnicity includes two categories: “Han” and “minority”; household registration includes two categories: “local county (district)” and “foreign county (district)”; birth weight includes three categories: “light,” “moderate” and “heavy”; and myopia includes two categories: “yes” and “no.”

The family level includes seven variables: only child, father’s education, mother’s education, family economic conditions, parental harmony, parental discipline, and stress due to parental educational expectations. Only child includes both “yes” and “no” categories. For the parents’ education level, referring to the study by Zhong et al. (80), “no education” and “primary school” are assigned as 1, that is, “primary school and below”; “junior school” is assigned as 2; “special/ technical school,” “vocational school” and “senior school” are assigned as 3, that is, “senior school/ secondary vocational”; and “junior college,” “bachelor’s degree” and “postgraduate student and above” are assigned as 4, that is, “college and above.” Family economic conditions were assigned using a 5-point scale, with 1 to 5 indicating very difficult to very rich. Parental harmony was judged using the declarative sentence “My parents have a good relationship with each other,” where “yes” was assigned a value of 1 and “no” was assigned a value of 2. This study used exploratory factor analysis to extract one composite indicator from eight question items reflecting the degree of parental discipline (homework/tests, performance at school, going to school every day, what time we go home every day, who we make friends with, how we dress, how long we spend on the Internet, and how long we spend watching TV), with higher values indicating stricter parental discipline. Stress due to parental educational expectations was assigned using a 5-point scale, with 1 to 5 indicating no stress to a lot of stress.

The school level included five variables: whether or not they had attended kindergarten, time spent on homework, time spent in tutoring classes, time spent on extracurricular physical activity, and whether or not they boarded. For homework, tutoring and extracurricular physical activity time, CEPS investigated the hours and minutes of average time spent by students in the above activities on school days and rest days, which the study summed up into minutes of activity for the week and divided by seven to obtain the average daily activity time.

### 3.3 Mathematical statistics

This study used SPSS 25.0, Stata 16.0, and AMOS 24.0 software for data processing and statistical analysis. Descriptive statistics were used for continuous variables using means (*M*) and standard deviations (*SD*); and for categorical variables using frequencies and percentages. This study constructed multiple linear regression models for analysis. In which Chinese, Mathematics, English and overall performance were used as dependent variables, respectively, and screen time, television watching time, internet access and game playing time were used as independent variables, respectively. Heterogeneity was indicated if the regression coefficients for one item of time spent watching television and time spent surfing the internet and playing games were not significant or if the regression coefficients for the two items were one positive and one negative. If both regression coefficients were homoscedastic and significant, the difference in regression coefficients was calculated using the *Z*-test. The *Z*-test was calculated as *Z* = $\frac{\beta 1-\beta 2}{\sqrt{{\mathrm{SE}}_{1^{2}}+{\mathrm{SE}}_{2^{2}}}}$ , where β1 and SE1 denote the regression coefficients and standard errors for time spent watching television, and β2 and SE2 denote the regression coefficients and standard errors for time spent surfing the internet and playing games. In this study, the Variance Inflation Factor (VIF) was used to detect multicollinearity among variables. It was found that the VIF values of all variables were less than 2, indicating that there was no significant multicollinearity among the variables. Additionally, a graphical method was employed to test for heteroscedasticity. By plotting a scatter diagram of the squared residuals against the sample data, it was observed that the scatter plot showed an irregular and random distribution, without any change in accordance with the variation of the sample data. Therefore, it can be concluded that the regression model does not exhibit heteroscedasticity. Finally, this study used total performance as the dependent variable, screen time as the independent variable, and cognitive performance, physical and mental health, and social constructs as the mediating variables in a structural equation modeling to explore the underlying mechanisms of the relationship between screen time and adolescents’ academic performance. CMIN/DF, RMSEA, NFI, RFI, IFI, TLI and CFI values are used to test the model fitness, in which the CMIN/DF value between 1 and 3 indicates a good model fit; the RMSEA value between 0.08 and 0.10 indicates an ordinary fit, and the value between 0.05 and 0.08 indicates a reasonable fit, while the value less than 0.05 indicates a very high fit; the NFI, RFI, IFI, TLI, and CFI values are mostly between 0 and 1, in which the closer to 1 the better the model fit. The values of NFI, RFI, IFI, TLI and CFI are mostly between 0 and 1, the closer to 1 the better the model fit, where the values of TLI, CFI and IFI may be greater than 1 (81).

## 4 Results

### 4.1 Basic information about the participants

The mean scores of 17,150 students were (84.13 ± 19.99), (79.88 ± 31.02), and (80.92 ± 29.91) in Chinese, Mathematics, and English, respectively, and the mean score of the total scores of the three subjects was (244.93 ± 72.58); and the mean daily screen time, TV watching time, and internet surfing and game playing time were (100.22 ± 95.32) min, (58.84 ± 63.37) min and (41.39 ± 59.50) min, respectively. The participants were equally divided between males and females, including 52.7% of seventh-grade students and 47.3% of ninth-grade students, including 91.9% of Han students and 8.1% of minority students, 82.6% of whom had their household registrations in their counties (districts), 43.7% of whom were only children, 80.3% of whom attended kindergarten, and 31.5% of whom boarded on school days (Table 1).

**Table 1:** Demographic information of the participants.

Variable	Category	Num.	Percentage (%)
Grade	Seventh-grade	9,030	52.7%
	Ninth-grade	8,120	47.3%
Gender	Boy	8,567	50.0%
	Girl	8,583	50.0%
Ethnicity	Han	15,766	91.9%
	Manority	1,384	8.1%
Household registration	Local	14,168	82.6%
	Foreign	2,982	17.4%
Only child	Yes	7,493	43.7%
	No	9,567	56.3%
Kindergarten	Yes	13,777	80.3%
	No	3,373	19.7%
Board	Yes	5,400	31.5%
	No	11,750	68.5%

### 4.2 The relationship between screen time and academic performance

After controlling for relevant variables, the results of the multiple linear regression analysis (Table 2) of screen time and adolescents’ academic performance showed that there was a significant negative correlation (β = −0.113 to −0.022, *p* < 0.01) between screen time and adolescents’ academic performance (Models 1 to 4), and research hypothesis H1 was supported. Model 2 had the largest estimated coefficient (β = −0.048), followed by Model 3 (β = −0.043), and finally Model 1 (β = −0.022), indicating that there is the largest negative association between screen time and adolescents’ mathematics scores, followed by English scores, and finally Chinese scores. That is to say, for every additional minute of screen time, adolescents’ overall academic performance will decrease by 0.113 points, their mathematics scores will decrease by 0.048 points, their English scores will decrease by 0.043 points, and their Chinese scores will decrease by 0.022 points.

**Table 2:** Multiple linear regression analysis of screen time and adolescents’ academic performance.

Variable	Model 1 Chinese	Model 2 Mathematics	Model 3 English	Model 4 Overall
Screen time	−0.022**	−0.048**	−0.043**	−0.113**
Grade	7.557**	1.348**	−7.855**	1.050
Gender	6.502**	1.452**	10.914**	18.867**
Ethnicity	1.204*	0.594	4.311**	6.109**
Household registration	3.237**	3.477**	2.008**	8.722**
Birth weight				
Moderate	0.829*	0.428	0.115	1.372
Heavy	1.926**	1.420	1.105	4.451*
Myopia	−4.459**	−7.042**	−6.331**	−17.823**
Only child	−2.902**	−5.524**	−5.608**	−14.034**
Father’s education				
Junior school	1.645**	1.568*	2.687**	5.900**
Senior school/secondary vocational	4.214**	4.882**	6.333**	15.429**
College and above	5.786**	9.476**	10.450**	25.713**
Mother’s education				
Junior school	2.217**	2.618**	3.051**	7.885**
Senior school/secondary vocational	3.713**	4.968**	6.474**	15.155**
College and above	4.408**	8.563**	9.336**	22.307**
Mother’s education	2.335**	2.420**	2.691**	7.446**
Parental harmony	−0.284	−2.282**	−1.074	−3.639**
Parental discipline	0.761**	0.186	1.032**	1.979**
Stress due to parental educational expectations	−0.735**	−2.010**	−1.582**	−4.327**
Whether or not they had attended kindergarten	−1.922**	−4.040**	−3.720**	−9.682**
Time spent on homework	0.016**	0.032**	0.025**	0.733**
Time spent in tutoring classes	−0.004	−0.017**	−0.008	−0.287*
Time spent on extracurricular physical activity	0.004	0.007	0.000	0.011
Whether or not they boarded	−1.373**	−3.212**	−0.351	−4.936**

*P < 0.05; **P < 0.01.

In addition, at the individual level, adolescents who were female (β = 18.867), minority (β = 6.109), foreign county (β = 8.722), and heavier birth weight (β = 4.451) were correlated with higher academic performance (*p* < 0.05) (Model 4). Students who were not myopic had lower academic performance (β = −17.823, *p* < 0.01) compared to those who were myopic (Model 4). Ninth graders were significantly higher than seventh graders in language (β = 7.557) and mathematics (β = 1.348) scores (*p* < 0.01), and significantly lower (β = −7.855, *p* < 0.01) than seventh graders in English scores (Models 1 to 3). At the family level, being a non-only child (β = −14.034) and having a dysfunctional parental relationship (β = −3.639) were associated with lower academic performance among adolescents (*p* < 0.01) (Model 4). Parental education was positively correlated with adolescents’ academic performance (*p* < 0.01), that is, as parental education increased, adolescents’ academic performance also increased (Model 4). Family economic conditions (β = 7.446) and degree of parental discipline (β = 1.979) were positively correlated with adolescents’ academic performance (*p* < 0.01); and the stress due to parental educational expectations was negatively correlated with adolescents’ academic performance (β = −4.327, *p* < 0.01) (Model 4). At the school level, not having attended kindergarten (β = −9.682) and not boarding at school (β = −4.936) were associated with lower academic performance among adolescents (*p* < 0.01) (Model 4). Time spent on homework was positively correlated with adolescents’ academic performance (β = 0.733, *p* < 0.01), time spent on tutorial classes was negatively correlated with adolescents’ academic performance (β = −0.287, *p* < 0.05), and time spent on extracurricular physical activity had a non-significant relationship with adolescents’ academic performance (β = 0.011, *p* = 0.264) (Model 4).

### 4.3 The heterogeneity of the relationship between screen time and academic performance

After controlling for relevant variables, time spent watching television and time spent surfing the internet and playing games were both significantly negatively correlated with adolescents’ academic performance (*p* < 0.01), with time spent surfing the internet and playing games producing greater negative effects than time spent watching television on Chinese, Mathematics, English, and overall performance (Table 3). Given that the regression coefficients for the different types of screen time and adolescents’ academic performance were all negative and significant; the only way to explore the consistency of the relationship between type of screen exposure and academic performance was through a *Z*-test. The SEs for the relationship between time on watching television and adolescents’ Chinese, Mathematics, English, and overall performance were 0.002, 0.004, 0.003, and 0.008, respectively; and the SEs for the relationship between time on the internet and playing games and adolescents’ Chinese, Mathematics, English, and overall performance were 0.002, 0.004, 0.004, and 0.009, respectively. This study calculated by substituting β and SE into the formula that there was no significant difference between the regression coefficients of Chinese scores (*Z* = 0.354, *p* = 0.363) but there was a significant difference between the regression coefficients of Mathematics (*Z* = 1.945, *p* = 0.026), English (*Z* = 2.400, *p* = 0.008) and overall performance (*Z* = 1.993, *p* = 0.023). Thus, there was heterogeneity in the type of screen exposure in the relationship between screen time and adolescents’ academic performance, with time spent surfing the internet and playing games having more detrimental effects on adolescents’ Mathematics (β = −0.063), English (β = −0.047), and Overall performance (β = −0.148). Research hypothesis H1 was further supported.

**Table 3:** Multiple linear regression analysis of different screen exposure times and adolescents’ academic performance.

Variable	Model 5 Chinese	Model 6 Mathematics	Model 7 English	Model 8 Overall	Model 9 Chinese	Model 10 Mathematics	Model 11 English	Model 12 Overall
Time spent on watching television	−0.026**	−0.052**	−0.045**	−0.123**				
Time spent on the internet and playing games					−0.028**	−0.063**	−0.047**	−0.148**
Grade	7.483**	1.229**	−7.952**	0.759	7.756**	1.820**	−7.431**	2.165*
Gender	6.802**	2.093**	11.491**	20.385**	6.473**	1.330**	10.788**	18.591**
Ethnicity	1.307*	0.810	4.504**	6.621**	1.173*	0.508	4.227**	5.908**
Household registration	3.014**	3.004**	1.585**	7.603**	3.289**	3.630**	2.159**	9.078**
Birth weight								
Moderate	0.819*	0.415	0.106	1.340	0.873*	0.521	0.199	1.592
Heavy	1.962**	1.499	1.176	6.438*	1.931**	1.423	0.105	4.458*
Myopia	−4.420**	−6.988**	−6.290**	−17.698**	−0.426**	−7.394**	−6.647**	−18.664**
Only child	−2.890**	−5.524**	−5.614**	−14.027**	−3.039**	−5.813**	−5.865**	−14.717**
Father’s education								
Junior school	1.624**	1.524*	2.648**	5.796**	1.657**	1.597*	2.714**	5.968**
Senior school/secondary vocational	4.220**	4.920**	6.375**	15.515**	4.357**	5.179**	6.958**	16.133**
College and above	5.908**	9.781**	10.737**	26.426**	6.020**	9.938**	10.854**	26.812**
Mother’s education								
Junior school	2.102**	2.385**	2.845**	7.332**	2.301**	2.817**	3.235**	8.353**
Senior school/secondary vocational	3.591**	4.737**	6.275**	14.603**	3.898**	5.378**	6.847**	16.124**
College and above	4.39**	8.461**	9.256**	22.056**	4.669**	9.122**	9.838**	23.629**
Mother’s education	2.138**	2.014**	2.330**	6.482**	2.439**	2.679**	2.934**	8.052**
Parental harmony	−0.465	−2.666**	−1.418*	−4.550**	−0.241	−2.156**	−0.951	−3.349*
Parental discipline	0.940**	0.584*	1.395**	2.918**	0.837**	0.307	1.129**	2.273**
Stress due to parental educational expectations	−0.739**	−2.026**	−1.599**	−4.364**	−0.780**	−2.101**	−1.663**	−4.545**
Whether or not they had attended kindergarten	−1.875**	−3.969**	−3.663**	−9.507**	−2.084**	−4.387**	−4.032**	−10.503**
Time spent on homework	0.017**	0.035**	0.028**	0.081**	0.016**	0.033**	0.026**	0.074**
Time spent in tutoring classes	−0.005	−0.018**	−0.009	−0.031*	−0.004	−0.016**	−0.007	−0.027*
Time spent on extracurricular physical activity	0.004	0.006	−0.001	0.009	0.002	0.004	−0.002	0.004
Whether or not they boarded	−1.583**	−3.685**	−0.782	−6.049	−1.480**	−3.392**	−0.498	−5.370**

*P < 0.05; **P < 0.01.

### 4.4 The mechanisms of the relationship between screen time and academic performance

In this study, the path analysis was conducted using screen time as the independent variable, overall performance in three subjects as the dependent variable, and BMI, subjective health perception, sleep duration, mental health, peer relationships, parent–child relationships, and cognitive performance as the mediating variables. The results of the model fit test showed that CMIN/DF = 4.127, RMSEA = 0.013, and the values of NFI, RFI, IFI, TLI, and CFI were 0.988, 0.986, 0.999, 0.990, and 0.999, respectively, resulting in an overall good model fit. In this study, the coefficient values of the 21 paths were estimated using great likelihood, and the results (Table 4) showed that the coefficient values of the remaining 15 paths reached significance (*p* < 0.01), except for paths 1, 2, 10, 16, 17, and 19. This suggests that the pathway of screen time acting on cognitive and academic performance through BMI and subjective health perceptions does not hold, the pathway of screen time acting on academic performance through mental health does not hold, and the pathway of screen time acting on academic performance through Pathway 10 does not hold. Therefore, research hypotheses H2a and H2c were supported and research hypothesis H2b was partially supported.

**Table 4:** Table of path analysis coefficients for screen time and adolescents’ academic performance.

Num.	Pathway	*b*	β	*SE*	*CR*	*P*
1	Screen time → BMI	<0.001	−0.002	<0.001	−0.221	0.825
2	Screen time → Subjective health perception	<0.001	−0.006	<0.001	−0.727	0.467
3	Screen time → Sleep duration	0.035	0.046	0.006	6.302	<0.001
4	Screen time → Mental health	0.001	0.069	<0.001	9.022	<0.001
5	Screen time → Peer relationships	−0.001	−0.088	<0.001	−11.512	<0.001
6	Screen time → Parent–child relationships	−0.001	−0.071	<0.001	−9.293	<0.001
7	Screen time → Cognitive performance	−0.005	−0.125	<0.001	−16.518	<0.001
8	BMI → Cognitive performance	0.023	0.020	0.009	2.653	0.008
9	Subjective health perception → Cognitive performance	0.094	0.022	0.033	2.814	0.005
10	Sleep duration → Cognitive performance	−0.001	−0.014	<0.001	−1.775	0.076
11	Mental health → Cognitive performance	−0.226	−0.060	0.031	−7.281	<0.001
12	Peer relationships → Cognitive performance	0.425	0.090	0.037	11.537	<0.001
13	Parent–child relationships → Cognitive performance	0.110	0.029	0.030	3.718	<0.001
14	Screen time → Academic performance	−0.103	−0.135	0.005	−20.050	<0.001
15	Cognitive performance → Academic performance	7.868	0.403	0.132	59.616	<0.001
16	BMI → Academic performance	0.144	0.006	0.149	0.964	0.335
17	Subjective health perception → Academic performance	0.256	0.003	0.579	0.442	0.658
18	Sleep duration → Academic performance	−0.108	−0.109	0.007	−16.081	<0.001
19	Mental health → Academic performance	0.122	0.002	0.538	0.227	0.821
20	Peer relationships → Academic performance	11.461	0.124	0.640	17.902	<0.001
21	Parent–child relationships → Academic performance	2.020	0.028	0.513	3.935	<0.001

**Figure 3:**
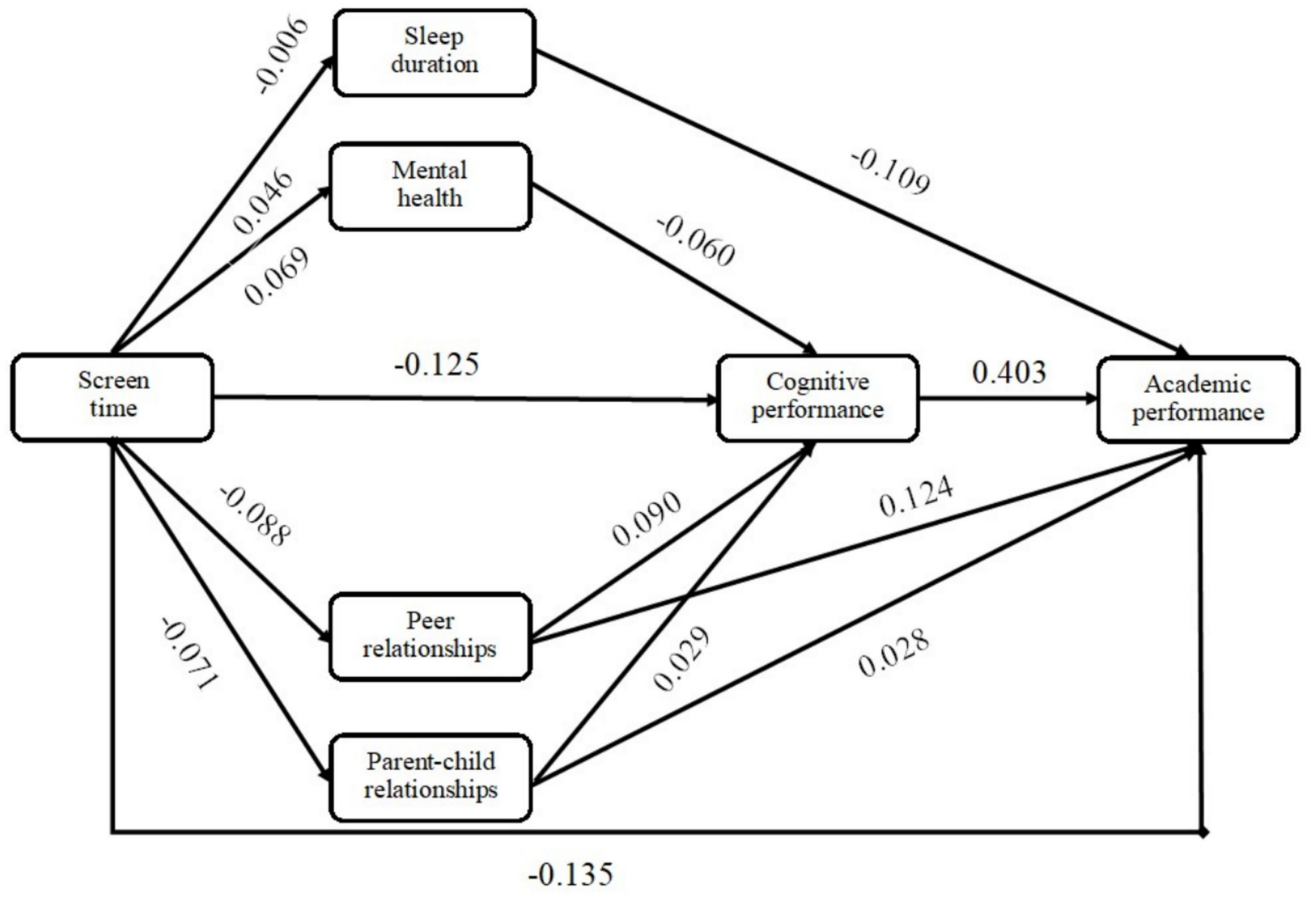
Mechanisms of the relationship between screen time and adolescents’ academic performance.

This study plotted the path analysis model based on the standardized regression coefficient values for the existence of significance among the variables. The results (Figure 3) showed that the direct effect sizes of screen time on sleep duration, mental health, peer relationships, parent–child relationships, and cognitive performance were 0.046, 0.069, −0.088, −0.071, and −0.125, respectively; the direct effect sizes of mental health, peer relationships, and parent–child relationships on cognitive performance were −0.060, 0.090, and 0.029, respectively; the direct effect sizes of sleep time, peer relationships, parent–child relationships, and cognitive performance on overall performance were −0.109, 0.124, 0.028, and 0.403, respectively; and screen time had a direct effect size of −0.315 on overall performance. In addition, the indirect effect size of screen time acting through sleep duration on overall performance was 0.046 × −0.109 = −0.005, and similarly, the indirect effect sizes of screen time acting through peer relationships, parent–child relationships, and cognitive performance were −0.011, −0.002, and −0.050, respectively; and the indirect effect sizes of screen time acting through Path 11, Path 12, and Path 13 were −0.002, −0.003, and −0.001, respectively. Thus, the total effect size of screen time acting on overall performance through cognitive performance was −0.056, and the total effect size of screen time acting on adolescents’ overall performance was −0.209. In addition, the effect sizes of screen time on overall performance through physical and mental health were −0.007 and through social constructs were −0.017, suggesting that social relationship constructs play a stronger mediating role in the relationship between screen time and academic performance.

## 5 Discussion

### 5.1 The relationship between screen time and academic performance

This study places the real-world issue of “screen time and adolescents’ academic performance” in the context of education in China, and the findings further support the idea of “negative impact.” Increased screen time means less time spent sleeping and being physically active, which in turn reduces the positive benefits of both on cognition and academic performance (43, 44, 62). In addition, increased screen time may lead to decreased time for social interaction and educational activities, directly impairing academic performance (42). In addition, according to the perspective of cognitive load theory, individuals will deplete cognitive resources when completing cognitive and behavioral tasks (82). In this study, when adolescents spend a large amount of time on screens, their cognitive resources are dispersed, and their cognitive abilities such as attention and memory for learning are weakened, which in turn leads to a decline in academic performance (82).

Sweetser et al. (27) have argued that the type of screen exposure is inconsistently related to adolescents’ cognitive and academic performance, categorizing television watching as negative screen time and internet surfing and game playing as positive screen time given the presence or absence of screen interaction. Adelantado-Renau et al. (23) and Hu et al. (83) have also concluded that passive television watching has somewhat more detrimental effects. However, the results of this study showed that watching television, surfing the internet, and playing games were all negatively associated with adolescents’ academic performance, and that more negative effects were associated with time spent surfing the internet and playing games. This study suggests that this may be related to the fact that Chinese adolescents face a greater academic burden. The pressure and maladaptation caused by the academic burden will prompt adolescents to use the internet and play games as a means of leisure and to cope with academic pressure, which in the long run will easily lead to the problem of internet addiction and mobile phone dependence (84, 85).

Some studies (86, 87) shows that the prevalence of internet and game addiction among Chinese adolescents is about 10 and 27.5%; the tendency of mobile phone dependence among urban middle and primary students is 23 and 11%, and higher in rural areas (30 and 17%). Xiong et al. (88) also showed that the prevalence of mobile phone addiction among children and adolescents in Eastern countries under heavier academic burdens was higher than in Western countries (29.8% vs. 18.4%). Whereas internet addiction and mobile phone dependence are associated with frontal, parietal, and hippocampal gyrus damage in individuals (89), there is a correlation with declines in cognitive performance such as executive function (90), which may lead to lower academic performance. In addition, while some studies have shown that e-sports or video games promote activation in prefrontal regions (91), excessive screen task processing will impair cognitive performance (26). Therefore, in the educational context of higher academic burden in China, time spent on the internet and playing games has more adverse effects on adolescents’ academic performance.

### 5.2 The mechanisms underlying the relationship between screen time and adolescents’ academic performance

This study explored the mechanisms underlying the relationship and found that cognitive performance was the primary pathway in the relationship, with sleep duration, mental health, peer relationships, and parent–child relationships being important distal pathways. Cognitive performance is considered to be the most important predictor variable of academic performance in adolescents (38, 47), and attention, thinking, memory, and executive functions intersect to varying degrees with computation and reading in terms of activation in task-processing brain regions (92, 93). In addition, there are associations between attention quality, executive function, and focused attention and self-control in classroom learning (94, 95), which have a positive effect on optimizing learning behavior and improving learning efficiency. However, excessive screen exposure can impair individual cognitive function, leading to learning inefficiencies, which in turn can have a negative effect on adolescents’ academic performance. The results of this study are similar to those of previous studies (17, 52–54).

In addition, on the distal pathway, this study found that social constructs mediated the relationship between screen time and academic performance more than physical and mental health. The reasons for this are analyzed in the following areas. Firstly, the construction of social relationships may be more sensitive than improvements in physical and mental health. For example, peer interaction, group identity and positive emotional experiences can be achieved through a few group games, whereas physical and mental health may require sustained exercise to achieve positive results. Secondly, a good social relationship implies that the individual has higher executive function and self-control, and is therefore more strongly linked to academic performance. O’Toole et al. (96) demonstrated that executive function is related to an individual’s self-regulation, effective problem solving, and social environmental adaptation. Deficits in executive function may increase the risk of impulsivity and aggressive behavior in adolescents and disrupt the establishment of good social relationships (96). Thirdly, multiple indicators of physical and mental health were not mediators of the relationship, reducing the estimated effect size of the effect. The results of this study are similar to the results of previous studies (28, 97), which have shown that the relationship between BMI and academic performance is not significant. Even a study (98) has shown that frailty is associated with lower academic achievement, which may be related to the replacement of the educational activities that students originally participated in with other activities. Fourthly, due to the limitations of the database, many physical and mental health variables that may be associated with screen time and academic performance have not been addressed, such as cardiorespiratory fitness, sleep quality, etc., and are expected to be further explored in subsequent studies.

### 5.3 Analysis of relevant control variables

Firstly, heavier birth weight is associated with higher academic performance. Solis-Urra et al. (99) showed that higher birth weight was associated with increased gray matter volume in the middle frontal gyrus, middle temporal gyrus, and bilateral cerebellum in adolescents, predicting the possibility of higher cognitive and academic performance (100). Secondly, non-myopic students have lower academic performance. This result is similar to the study by Cheng et al. (98), where myopia problems positively associated with academic performance may be caused by the accumulation of near learning activities. However, the sacrifice of visual health is not a necessary condition for improved grades, and severe myopia problems can cause mental problems such as anxiety and depression, which in turn can impair academic performance (98). Thirdly, there is a negative correlation between the time spent in tutorial classes and academic performance. It is difficult for students to internalize newly acquired knowledge in a short period of time, and blindly attending tutorial classes increases students’ academic burden while reducing the time available for internalization of the knowledge imparted by the school, which ultimately leads to the result of “the more tutorials, the worse the performance” (101). Finally, the relationship between the time spent on extracurricular physical exercise and academic performance was not significant. The reason for this is that there is an “inverted U-shaped” relationship between physical exercise and academic performance (28, 102) and a simple linear correlation ignores the intrinsic nature of the relationship.

## 6 Limitations of this study

Although this study has revealed the relationship and mechanisms between screen time and adolescents’ academic performance, there are still the following limitations. Firstly, this study is based on cross-sectional data, which can only reveal the correlation between screen time and academic achievement, but cannot determine the causal relationship. Therefore, we look forward to further verifying the causal mechanisms through longitudinal tracking data in the future. Secondly, screen time relies on students’ self-reports, which may be subject to recall bias, thereby affecting the accuracy of the research results. We hope that future studies will be based on more objective data (for example, monitoring devices’ internet traffic through routers or network management systems to identify high-bandwidth screen activities such as videos and games) for research. In addition, due to the limitations of the CEPS dataset, this study is unable to explore the relationship between the content of what adolescents watch on screens and their academic performance. Future studies is expected to adopt strict standards for classifying screen content to investigate the relationship between screen content, screen time, and adolescents’ academic performance, further enriching the research in this field. Thirdly, this study has confirmed some of the mediating paths proposed in the research hypothesis, but some mediating paths have not been confirmed, and there are inconsistencies with the results of previous studies. Therefore, further verification by subsequent studies is still needed. In addition, we also look forward to future studies exploring the extensive moderating factors of this relationship, so as to provide more refined suggestions for relevant policy-making. Finally, this study is based on the Chinese educational context, and cultural characteristics such as high academic pressure and widespread after-school tutoring among students may make the results difficult to generalize to other countries or regions.

### 6.1 Suggestions for practical application and future intervention studies

The adverse effects of screen exposure on adolescents’ cognitive and academic performance should be emphasized. (1) Schools should minimize online homework and reduce students’ exposure to screen media. In addition, schools should improve the quality of after-school homework, prevent students from blindly attending tutorial classes, optimize the time allocated for academic subjects, and provide students with adequate time for physical exercise, so as to promote students’ physical and mental health and enhance friendships among classmates. (2) Parents can provide their children with non-smartphones (i.e., phones that can only make calls) or set up youth modes on smartphones to restrict children’s exposure to screen media. Parents should supervise their children’s completion of online homework, prevent them from watching television, surfing the internet, and playing games on excuses, and ensure that their children get enough and good sleep; they should also take the lead in controlling their own screen time; they should pay attention to fostering their children’s extracurricular interests, and get involved in their children’s extracurricular activities, so as to promote the warming up of parent–child relationships. (3) Adolescents themselves should refrain from excessive exposure to screens, actively participate in extracurricular exercise activities, enhance their physical and mental health during exercise, and cultivate mutual help and friendship among classmates.

In addition, the main findings of this study have enlightening implications for future intervention studies. Firstly, future intervention studies should focus on promoting collaboration among families, schools, and society to form a combined force. Through the joint efforts of multiple stakeholders, a favorable environment can be created for adolescents to reduce screen time and improve academic performance. Secondly, community-based intervention projects can be carried out. By organizing educational activities, sports activities, and educational activities for parents, etc., it is possible to comprehensively prevent adolescents from spending excessive time on screens and promote their healthy growth.

## 7 Conclusion

This study used the CEPS survey data to explore the relationship between screen time and the academic performance of Chinese adolescents and its mechanisms. It was found that screen time was negatively related to adolescents’ academic performance, and that surfing the internet and playing games had a greater negative effect on academic performance. For the analysis of mechanisms, cognitive performance was found to be the main pathway in the relationship between screen time and adolescents’ academic performance, with sleep duration, mental health, peer relationships, and parent–child relationships acting directly or indirectly through the mediating role of cognitive performance on academic performance. The above findings have reference and guiding significance for the formulation of relevant policies and the implementation of measures to prevent excessive screen time.

## Data Availability

Publicly available datasets were analyzed in this study. This data can be found here: CEPS data can be downloaded on request by registering from the website http://ceps.ruc.edu.cn/. The data that support the findings of this study are available on request from the corresponding author upon reasonable request.
